# Supplementary material for: PyPlaque is an open-source python package for phenotypic analysis of virus plaque assays
Source: Sci Rep. 2025 Oct 16;15:36187. doi: 10.1038/s41598-025-20075-w (PMC12533079; doi:10.1038/s41598-025-20075-w)
Supplement: Supplementary file 1 — Supplementary Information. [file 41598_2025_20075_MOESM1_ESM.pdf]

Supplement to PyPlaque: an Open-source Python Package for Phenotypic Analysis of Virus Plaque Assays

August 1, 2025

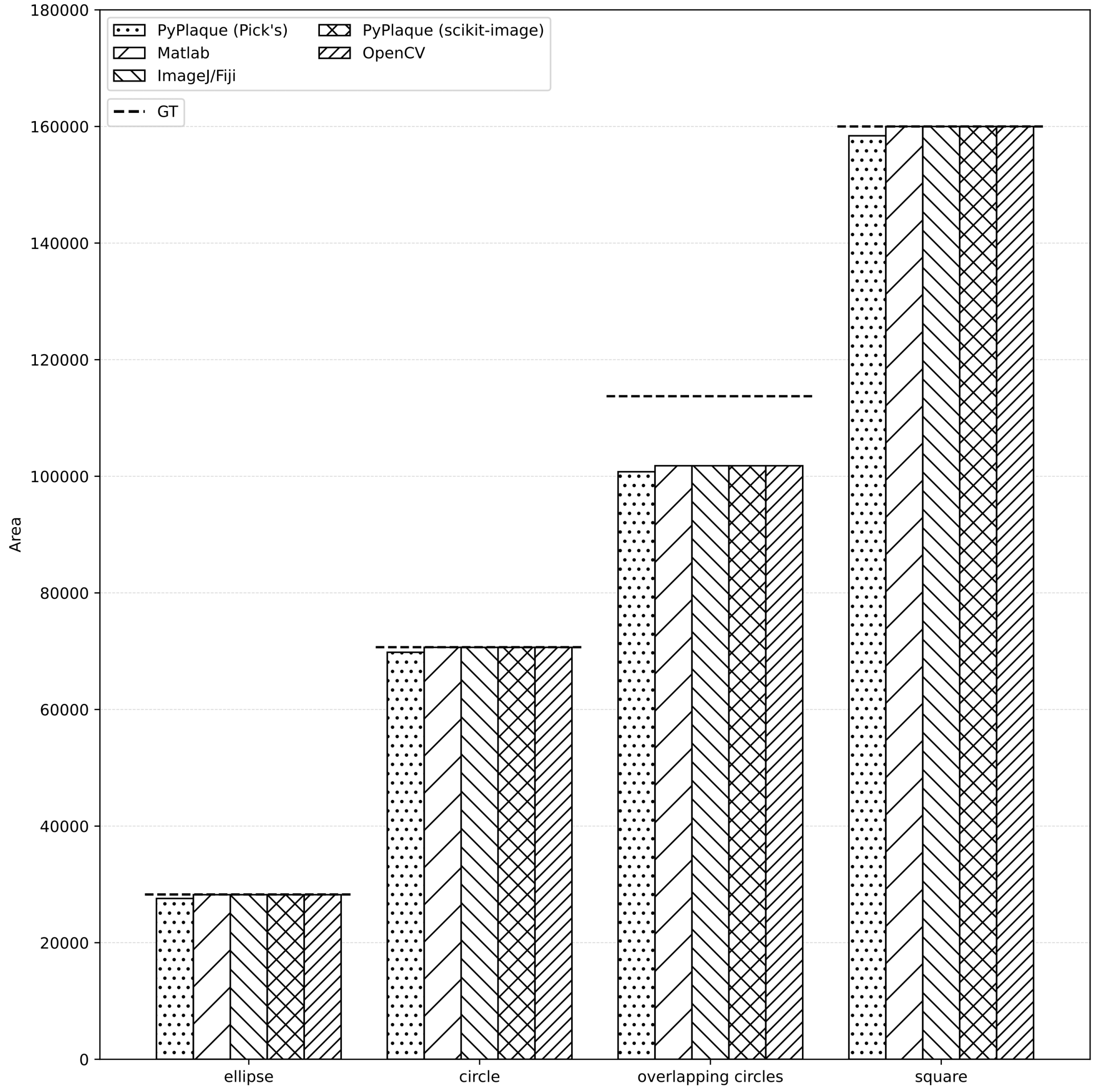

Figure S1: **Comparison between Area measurements of synthetic shapes between different bioimage analysis software.** This barplot is a visual representation of table 1 from the main paper.

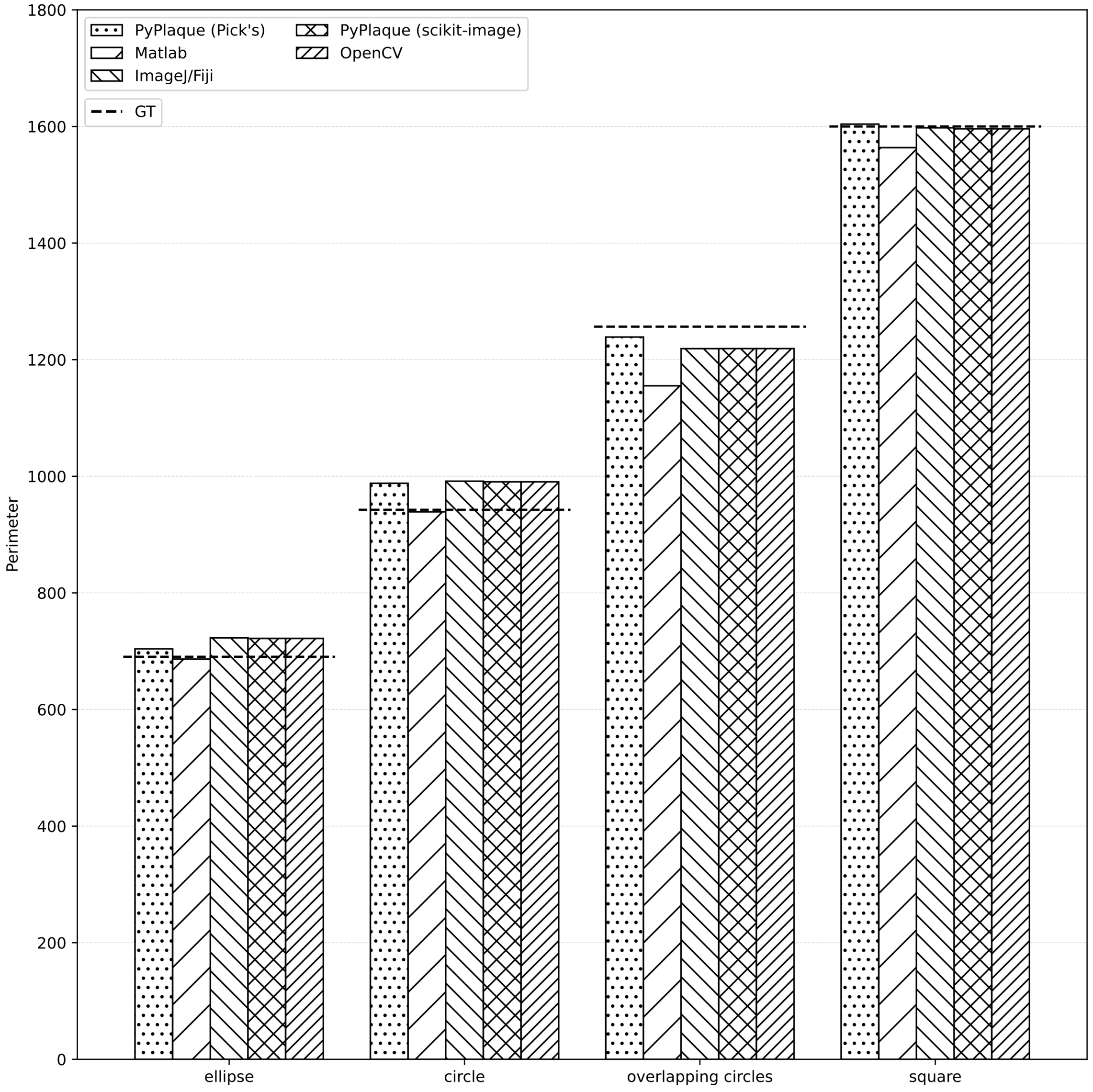

Figure S2: **Comparison between Perimeter measurements of synthetic shapes between different bioimage analysis software.** This barplot is a visual representation of table 2 from the main paper.
